# Functional Identification of the Dextransucrase Gene of *Leuconostoc mesenteroides* DRP105

**DOI:** 10.3390/ijms21186596

**Published:** 2020-09-09

**Authors:** Renpeng Du, Zhijiang Zhou, Ye Han

**Affiliations:** School of Chemical Engineering and Technology, Tianjin University, Tianjin 300072, China; durenpeng@tju.edu.cn (R.D.); zzj@tju.edu.cn (Z.Z.)

**Keywords:** *Leuconostoc mesenteroides*, complete genome sequence, dextransucrase, gene knockout, metabolic analysis

## Abstract

*Leuconostoc mesenteroides* DRP105 isolated from Chinese sauerkraut juice is an intensive producer of dextran. We report the complete genome sequence of *Leu. mesenteroides* DRP105. This strain contains a dextransucrase gene (*dsr*) involved in the production of dextran, possibly composed of glucose monomers. To explore the dextran synthesis mechanism of *Leu. mesenteroides* DRP105, we constructed a *dsr*-deficient strain derived from *Leu. mesenteroides* DRP105 using the Cre-loxP recombination system. The secondary structure prediction results showed that *Leu. mesenteroides* DRP105 dextransucrase (Dsr) was coded by *dsr* and contained 17.07% *α*-helices, 29.55% *β*-sheets, 10.18% *β*-turns, and 43.20% random coils. We also analyzed the dextran yield, monosaccharide change, organic acid, and amino-acid content of *Leu. mesenteroides* DRP105 and *Leu. mesenteroides* DRP105−*Δdsr*. The result showed that the lack of *dsr* changed the *Leu. mesenteroides* DRP105 sugar metabolism pathway, which in turn affected the production of metabolites.

## 1. Introduction

Exopolysaccharides (EPS) are used in medicine, food, and other industrial products due to their unique chemical, physical, and biological activities [1]. The diversity of EPS functions affects differences in chemical composition, in particular, the presence of different sugar residues, branching, chemical bond types, sugar modification, and chain length, all of which are determined through complex biosynthetic processes [2]. One of the most important examples of microbial EPS is glucan, as produced by *Leuconostoc mesenteroides*, which was found to have a wide range of applications in medicine. Sucrose is hydrolyzed by glucanase, and then the resulting d-glucosyl moiety is polymerized to produce glucan [3].

Given the wide commercial use of dextran, sufficient information must be obtained regarding the characteristics of EPS-producing strains [4]. The biosynthesis efficiency of lactic acid bacteria (LAB) glucan is low, and the synthesis process is complicated. Dextransucrase (Dsr) plays a decisive role in the structure of dextran. Studies show that Dsrs from different LABs have similar biochemical properties and structural characteristics [5]. However, these different Dsrs can form different glucan repeating units [6], which in turn affect the function of glucan. The mechanism of Dsr regulation during LAB glucan synthesis is not clear. Depending on the species, EPS from *Leuconostoc* have different structures. Genes that regulate dextran repeat unit synthesis, chain length, aggregation, and export are relatively conserved. To date, more than 30 confirmed dextransucrase gene (*dsr*) have been reported [7], and the catalytic performance of Dsr from different sources is very different, which means the corresponding Dsrs have many different morphologies.

To understand and optimize the production of EPS, the genetic information of the producing microorganism must be obtained. As no whole-genome sequence of *Leu. mesenteroides* DRP105 is publicly available, we sequenced the novel strain *Leu. mesenteroides* DRP105 isolated from Chinese sauerkraut juice [8]. The *dsr* gene as a nutritional selection marker has been studied for decades [7]. The classic strategy for obtaining dsr gene mutant strains is based on mutagenic screening and gene cloning techniques [9]. However, mutagenesis screening requires a long experiment time, is very laborious, and has a high probability of reverse mutation. Using gene cloning technology to construct overexpressed strains can only explore the strain metabolism of a single gene, but cannot systematically analyze the impact of other genes on the product production. Due to the versatility of the Cre-loxP recombination system, it is widely used for gene deletion and rearrangement in bacteria and eukaryotes [10,11]. However, there is no report regarding the deletion of the *dsr* gene in *Leu. mesenteroides*.

In this study, the gene *dsr* on *Leu. mesenteroides* DRP105 was removed from the genome through the Cre-loxP system. Then, we used the *dsr* gene to construct a food-grade expression vector, and studied the specific effect of the *dsr* gene on *Leu. mesenteroides* DRP105. These results help in the understanding of the underlying mechanism of EPS production of *Leu. mesenteroides* and facilitate relinking the genetic circuits involved in the production of dextran to optimize fermentation using the *Leu. mesenteroides* DRP105 strain.

## 2. Results and Discussion

### 2.1. General Genome Properties

**Figure 1:**
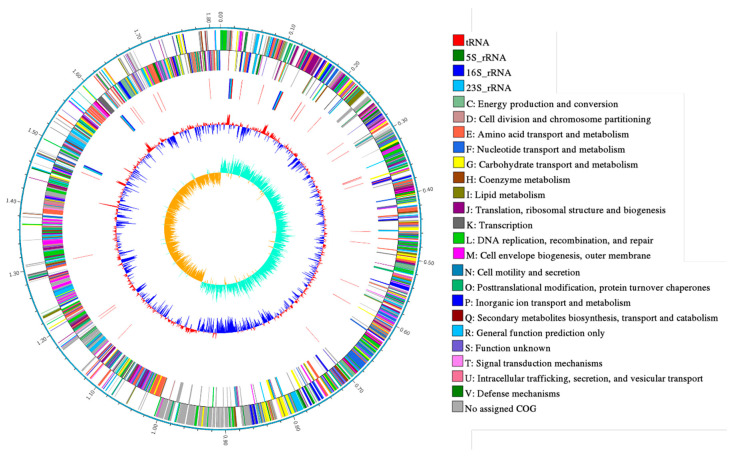
Circular genome map of *Leuconostoc mesenteroides* DRP105.

Statistics of PacBio raw data are shown in Table 1. The results of the circular genome map of *Leu. mesenteroides* DRP105 are shown in Figure 1. *Leu. mesenteroides* DRP105 has one circular chromosome of 1,815,345 bp, with 37.267% G + C content. This bacteria also contains five plasmids (named pLm-DRP01, pLm-DRP02, pLm-DRP03, pLm-DRP04, and pLm-DRP05 of 37,314 bp with 35.388% G + C, 13,823 bp with 36.743% G + C, 12,703 bp with 35.558% G + C, 12,404 bp with 34.425% G + C, and 12,703 bp with 37.967% G + C) (Table 2). The genome encodes 71 transfer RNA (tRNA) and 12 ribosomal RNA (rRNA) operons. Hong et al. determined a draft genome sequence of *Leuconostoc garlicum* KCCM 43211 with the same method and examined similar genomic features with a lactic acid bacterium [12].

**Figure 2:**
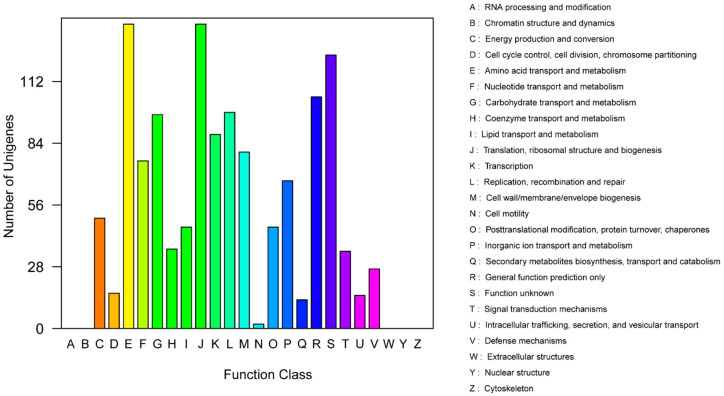
Clusters of orthologous groups (COG) annotation classification statistics of *Leu. mesenteroides* DRP105 genome.

**Figure 3:**
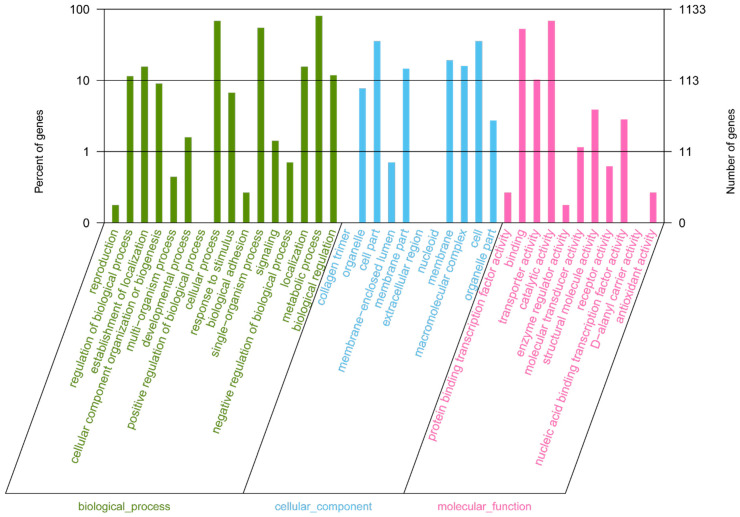
Gene Ontology (GO) annotation classification statistics of the *Leu. mesenteroides* DRP105 genome.

In the clusters of orthologous groups (COG) distribution, R (general function prediction only; 105 open reading frames (ORFs)), J (translation, ribosomal structure, and biogenesis; 138 ORFs), and E (amino acid transport and metabolism; 138 ORFs) were abundant categories. Genes responsible for replication, recombination, and repair (98 ORFs), carbohydrates (97 ORFs), and energy production and conversion (50 ORFs) were abundant among the SEED subsystem categories (Figure 2). We found 915 genes assigned to be involved in information metabolic processes, and another 253 genes were identified to participate in cellular processes [13]. The most abundant 1594 genes encoded the proteins for catalytic activity (Figure 3). Through Basic Local Alignment Search Tool (BLAST) comparison with the Swiss-Prot database, a total of 4487 proteins were annotated in *Leu. mesenteroides* DRP105, including a large number of proteases. A total of 130 pathways, including metabolic pathways, synthetic pathways, membrane transport, signal transmission, cell cycle, disease-related pathways, chemical molecules, enzymes, and enzymatic reactions, were collected using the Kyoto Encyclopedia of Genes and Genomes (KEGG) LIGAND database.

**Figure 4:**
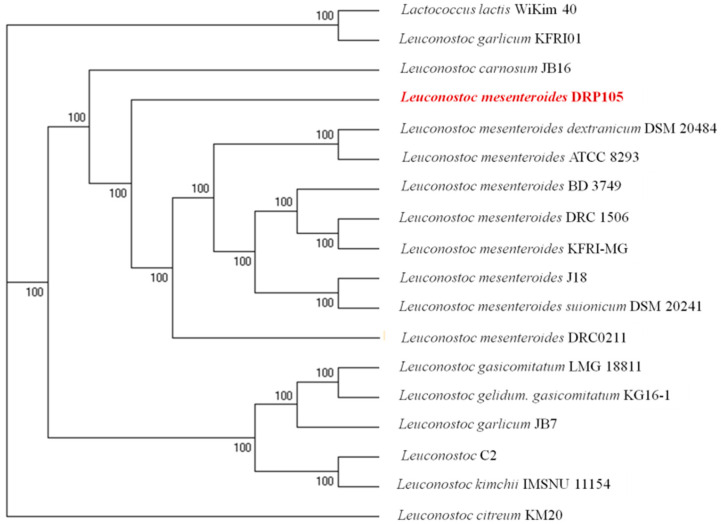
A molecular phylogenetic tree constructed using the maximum-likelihood method.

**Figure 5:**
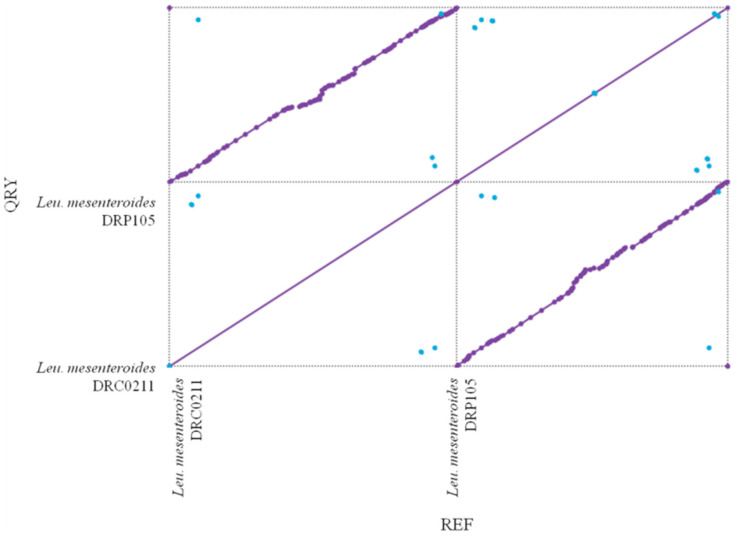
Collinearity analysis of *Leu. mesenteroides* DRP105 and *Leu. mesenteroides* DRC0211.

**Figure 6:**
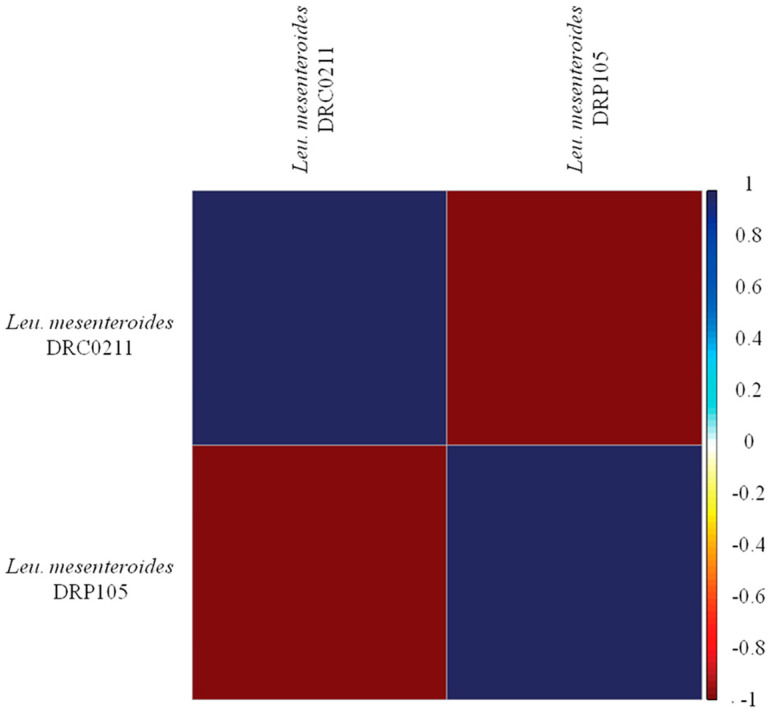
Average nucleotide identity (ANI) analysis of *Leu. mesenteroides* DRP105 and *Leu. mesenteroides* DRC0211.

The phylogenetic tree of strains was constructed on the basis of the single-copy gene method as shown in Figure 4. *Leu. mesenteroides* DRP105 is the closest to *Leu. mesenteroides* DRC0211. Therefore, follow-up advanced analysis compares *Leu. mesenteroides* DRP105 and *Leu. mesenteroides* DRC0211. The collinearity analysis of the genomic sequences of *Leu. mesenteroides* DRP105 and *Leu. mesenteroides* DRC0211 is shown in Figure 5. The two strains exhibited extreme collinearity. Only a few genomic inversion events occurred in the noncore region, indicating that these two strains consisted of relatively conserved genomic structures and homologous genes, and large-scale insertions, deletions, transformations, and translocations did not occur at the genomic level. The correlation of DNA was previously used as an indicator to identify new prokaryotes [14]. However, in this era of genomics, the average nucleotide identity (ANI) between a given pair of genomes is the first choice [15]. The ANI value between *Leu. mesenteroides* DRP105 and *Leu. mesenteroides* DRC0211 was greater than 91.87%, indicating that the two strains are the same species (Figure 6).

### 2.2. Gene Analysis of Dextransucrase

**Figure 7:**
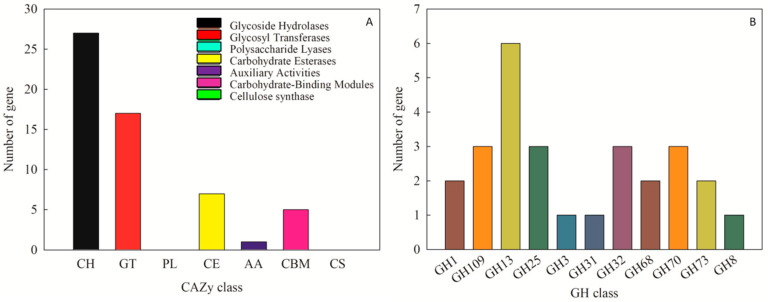
CAZy annotation of *Leu. mesenteroides* DRP105 ((**A**): CAZy annotation; (**B**): glycoside hydrolases (GH) family annotation).

The CAZy database is used to annotate carbohydrate-related enzymes, mainly including glycoside hydrolase glycosyl transferases, polysaccharide lyases, carbohydrate esterases, auxiliary activities, carbohydrate-binding modules, and cellulose synthase. In the complete genome sequence of *Leu. mesenteroides* DRP105, there were 140 proteins successfully annotated by the CAZy database, of which most were 27 glycoside hydrolases (GH) (Figure 7A). Among the glycoside hydrolases, three were annotated as the GH 70 family (Figure 7B). Among these genes, we found a *dsr* gene. The enzyme protein formed after translating *dsr* into amino acids was named Dsr. The analysis of secondary metabolites showed that no dextran gene cluster was found in the strain genome. This indicated that the dextran produced by *Leu. mesenteroides* DRP105 was a homopolysaccharide. During the synthesis of homopolysaccharides, Dsr was a secretase that could catalyze the synthesis of dextran from sucrose. This result was consistent with the Dsr annotated by the KEGG pathway analysis. Dsr belongs to the GH70 family and contains eight conserved regions [16]. Among them, COG5263 is the C-terminal repeat region of Dsr, which contains YG repeat units, and is responsible for carbohydrate transport and metabolism.

**Figure 8:**
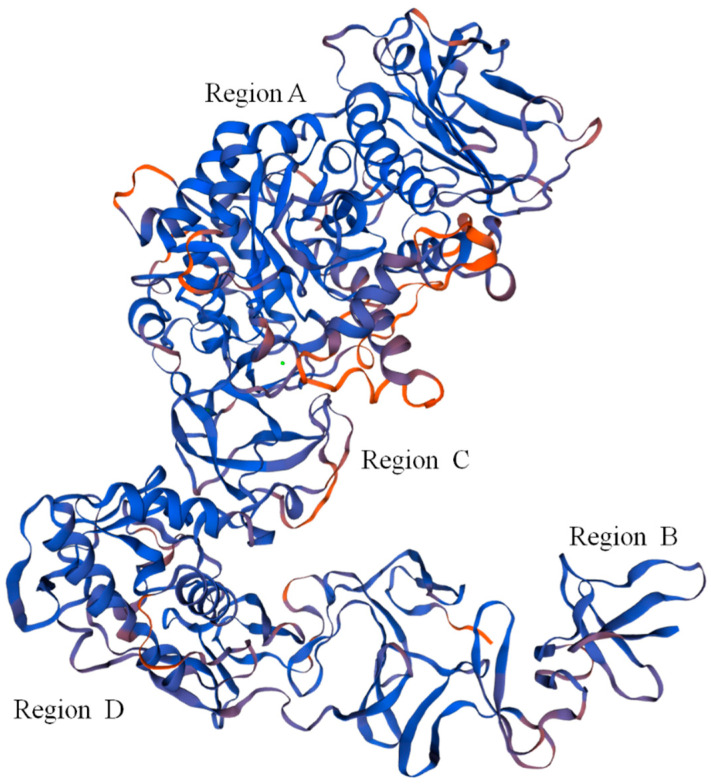
Predicted tertiary structure of dextransucrase (Dsr) translated from the *dsr* gene.

The Dsr protein coded by the *dsr* gene had the highest similarity to the *Leu. mesenteroides* Dsr (ABC75033.1), reaching 92.21%. The Dsr sequence contained 27 open reading frames (OFR), while the start codon was ATG and the stop codon was TGA. Repeat sequence analysis showed that Dsr has 34 repeat sequences. The theoretical molecular weight and theoretical isoelectric point of Dsr were 169,841.46 Da and 4.46, respectively. The phosphorylation site analysis showed that Dsr contains 99 Ser, 120 Thr, and 101 Tyr, which may be the phosphorylation site of the protein kinase. The Dsr mass instability index was 21.70, which denotes a stable protein. Predictive analysis of subcellular location found that 9.98% of Dsr exists outside the cell and 0.02% exists outside the cytoplasmic wall. Therefore, Dsr is a protein located outside the cell. Protein transmembrane region analysis indicated that Dsr contains a transmembrane region, and the protein may act as a membrane receptor. This result was consistent with the results of subcellular localization analysis. *Leu. mesenteroides* DRP105 Dsr secondary-structure prediction results showed that the protein contained 17.07% *α*-helices, 29.55% *β*-sheets, 10.18% *β*-turns, and 43.20% random coils. Monchois et al. used circular dichroism (CD) to identify the secondary structure of *Streptococcus downei* GTF-I Dsr, and found that *β*-sheets and random curls were the main secondary-structural components of Dsr [17]. The prediction result of the tertiary structure of Dsr translated from the *dsr* gene is shown in Figure 8. The Dsr contains four basic structural regions contained in conventional Dsr, N-composed *β*-sheet antiparallel structures, a terminal signal peptide region B, an α/β barrel-like N-terminal catalytic region (alternately composed of eight *α*-helices and eight *β*-sheets) (region A), a highly variable stretch region opposite to region B, adjacent to region A and C, and a C-terminal repeating unit region D related to glucan binding [16]. Domain A is the largest domain; it forms the catalytic core together with elements from domain B. Domain C is inserted between helix α_8_ and strand β_1_, and domain B connects strand β_3_ to helix α_3_. The three proposed catalytic residues (the nucleophilic aspartate, the acid/base glutamate, and the transition state stabilizing aspartate) lie at the bottom of a deep pocket in this domain [18,19]. This structure conforms to the conformation of other Dsrs.

### 2.3. The dsr Gene Deletion of Leu. mesenteroides DRP105

**Figure 9:**
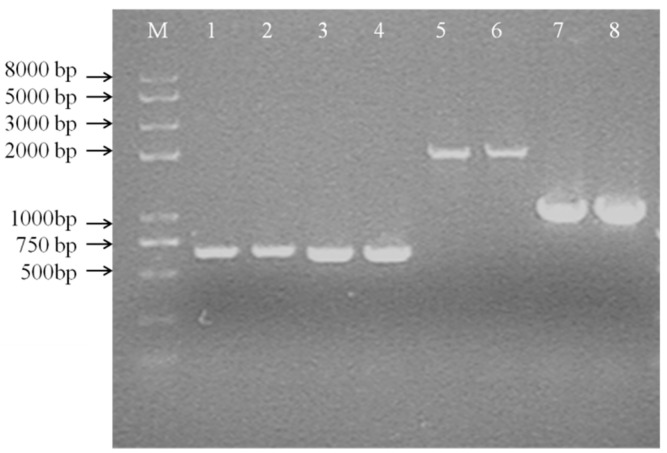
Colony PCR of the first crossover strain (A) (M: DNA Marker DL 8000; 1, 2: PCR product by Cm-F and Cm-R; 3, 4: PCR product by Ery-F and Ery-R; 5, 6: PCR product by *dsr*-up-F and Cm-R; 7, 8: PCR product by *dsr*-down-F and *dsr*-down-R).

When a single crossover occurred in *Leu. mesenteroides* DRP105, fragments could be amplified in PCR with both Ery and Cm primers. When the *dsr* gene was fully deleted, only one fragment could be amplified by Cm primers (Figure 9). The *dsr* gene fragments amplified using the bacterial genome as a template were of the same molecular weight as the target DNA (650 bp) with the Cm-F/Cm-R primers. In order to analyze whether the *dsr* gene was replaced by the *cm* gene cassette (1019 bp), the primers *dsr*-down-F/*dsr*-down-R were used to amplify the flanking regions of the Cm cassette (for *dsr*, the primer *dsr*-down-F for amplification of the 5′-region and the primer *dsr*-down-R for amplification of the 3′-region) (Figure 9).

PCR with the wild-type strain as a template yielded approximately 2.0-kb fragments, whereas the double crossover strain gave 1.0-kb fragments in PCR with the primers *dsr*-down-F/*dsr*-down-R. When the plasmid pNZTS-Cre was transformed into *Leu. mesenteroides* DRP105, remediated recombination was confirmed by PCR amplification with the primer *dsr*-1-F/*dsr*-1-R as above. These results suggested that the *dsr* gene was completely deleted in *Leu. mesenteroides* DRP105. As a selection maker, the *dsr* gene was as efficient as ermA was in *Leu. mesenteroides*, and it could be used as the nutrition selection marker in food-grade vectors. Zhu et al. determined that the gene *thyA* on the chromosome of *Lactococcus lactis* NZ9000 was deleted in vivo via the Cre-loxP system [20]. However, to date, there is no research regarding the *dsr* gene with the Cre-loxP recombination system. This was the first instance of reporting this result. This demonstrated that this method can effectively study gene regulation in the *Leu. mesenteroides* metabolic pathway.

### 2.4. Metabolic Analysis

**Figure 10:**
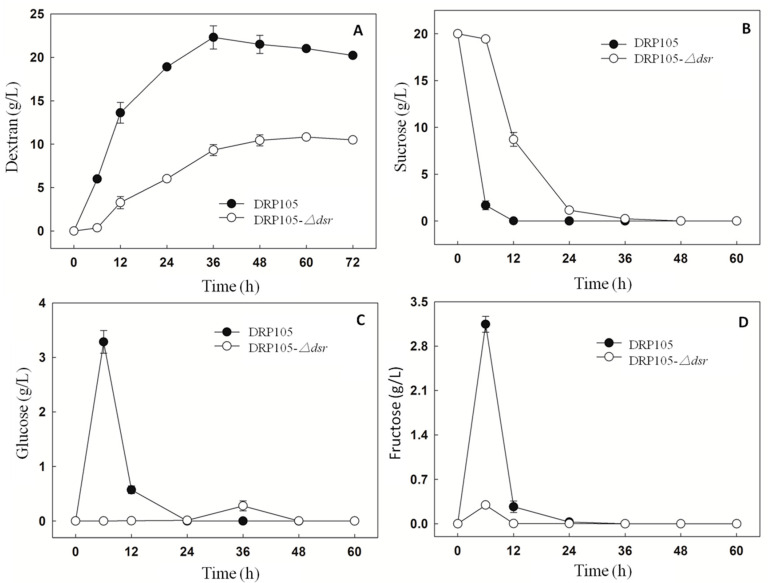
The carbohydrate content during *Leu. mesenteroides* DRP105 and *Leu. mesenteroides* DRP105−*Δdsr* fermentation ((**A**): dextran; (**B**): sucrose; (**C**): glucose; (**D**): fructose).

The sucrose metabolism and EPS production progress of *Leu. mesenteroides* DRP105 and *Leu. mesenteroides* DRP105−*Δdsr* are shown in Figure 10. At 6 h of fermentation, the *Leu. mesenteroides* DRP105 converted sucrose to glucose, while *Leu. mesenteroides* DRP105−*Δdsr* did not convert sucrose to glucose (Figure 10C). This result is similar to the metabolic process of many strains of *Leuconostoc* [21,22]. In the carbon metabolism pathway, Dsr catalyzes glucose to form glucose and fructose, and glucose polymerizes into dextran. As *Leu. mesenteroides* DRP105−*Δdsr* did not effectively use the sucrose, the sucrose content was significantly higher than that of *Leu. mesenteroides* DRP105 (*p* < 0.05) (Figure 10B), and the contents of glucose and fructose were significantly lower than that of *Leu. mesenteroides* DRP105 (Figure 10C,D), indicating that the *dsr* gene plays a key role in the metabolism of sucrose. As the fermentation time progressed, the sucrose content was gradually decreased in *Leu. mesenteroides* DRP105−*Δdsr*, and the dextran content gradually increased; however, it was always lower than that of *Leu. mesenteroides* DRP105 (*p* < 0.05) (Figure 10A). Therefore, we speculate that the *dsr* gene is key for the production of dextran by *Leu. mesenteroides* DRP105.

**Figure 11:**
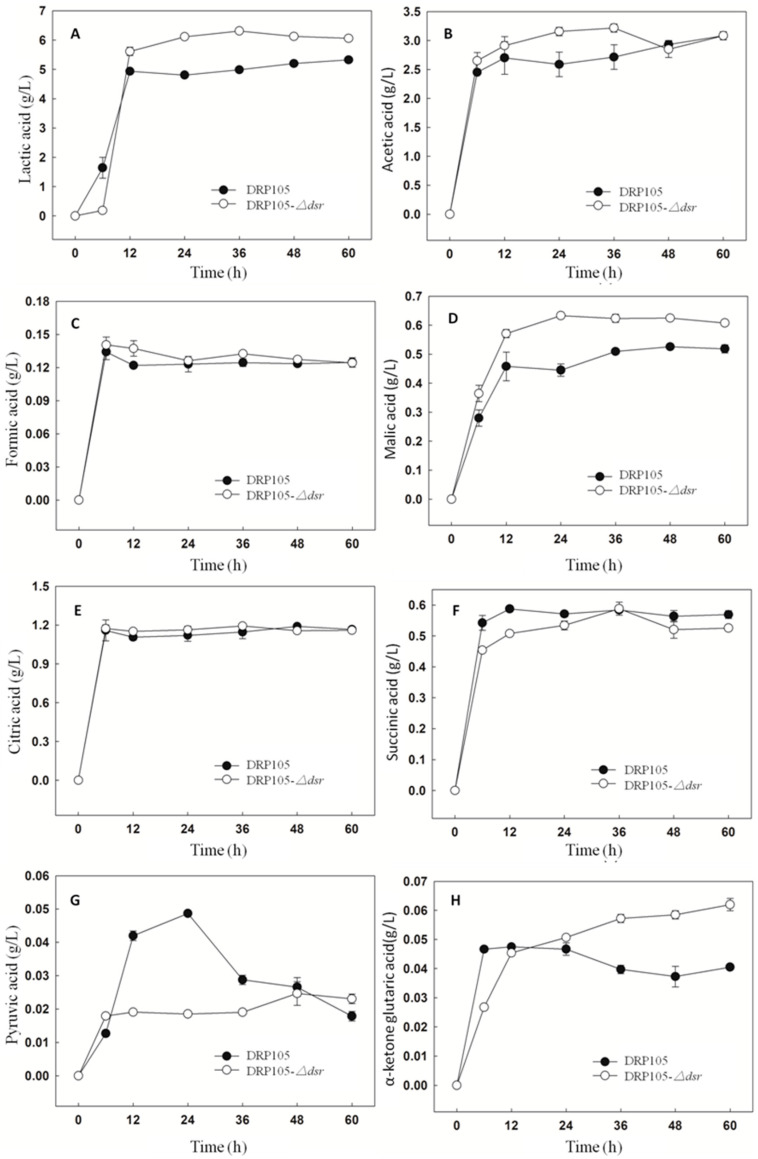
The organic acid content during *Leu. mesenteroides* DRP105 and *Leu. mesenteroides* DRP105−*Δdsr* fermentation ((**A**): lactic acid; (**B**): acetic acid; (**C**): formic acid; (**D**): malic acid; (**E**): citric acid; (**F**): succinic acid; (**G**): pyruvic acid; (**H**): *α*-ketone glutaric acid).

Figure 11 shows the amount of organic acids produced during the sucrose metabolism of *Leu. mesenteroides* DRP105 and *Leu. mesenteroides* DRP105−*Δdsr*. With the progress of fermentation time, the contents of lactic acid, acetic acid, formic acid, malic acid, citric acid, succinic acid, pyruvic acid, and α-ketoglutaric acid all showed a tendency to increase first and then stabilize. There was no significant difference in the content of the strains. After 6 h of fermentation, the organic acid content accumulated rapidly. The contents of acetic acid, malic acid, and pyruvic acid in *Leu. mesenteroides* DRP105−*Δdsr* were higher than those in *Leu. mesenteroides* DRP105 (*p* < 0.05), while the contents of lactic acid, succinic acid, and α-ketoglutaric acid were lower than those in wild bacteria (*p* < 0.05). At the end of fermentation, the contents of lactic acid, malic acid, and α-ketoglutaric acid in *Leu. mesenteroides* DRP105−*Δdsr* were higher than those in *Leu. mesenteroides* DRP105 (*p* < 0.05). During the whole fermentation process, the content of pyruvate and α-ketoglutarate of the two strains changed greatly. This result was similar to Zhang’s research [21]. Therefore, it can be seen that knocking out the *dsr* gene will affect the accumulation of organic acids in the strain. It may be that, in the process of the sucrose metabolism, different gene deletions cause changes in the sugar metabolism pathway, which affect the utilization of sugar and the production of products.

The content of 21 free amino acids in the fermentation broth at 24 h is shown in Table 3. Total amino acids in the fermentation broth of *Leu. mesenteroides* DRP105 and *Leu. mesenteroides* DRP105−*Δdsr* were 3329.7 ± 5.3 mg/kg and 3283.7 ± 3.8 mg/kg, respectively, with significant differences (*p* < 0.0001). Taurine, theanine, and hydroxyproline were not detected in the two strains. In *Leu. mesenteroides* DRP105−*Δdsr*, the contents of glycine, alanine, and cystine were slightly higher than those in wild bacteria, and the contents of other free amino acids were lower than those in wild bacteria (*p* < 0.05). Therefore, the results demonstrated that, due to the knockout of the *dsr* gene, the metabolic pathway of *Leu. mesenteroides* DRP105 was changed, which affected the change in free amino-acid content.

## 3. Materials and Methods

### 3.1. Strains and Culture

*Leu. mesenteroides* DRP105 was isolated from Chinese sauerkraut juice [8]. This strain was routinely grown in Man Rogosa Sharpe (MRS) medium (glucose 20 g/L, tryptone 10 g/L, beef extract 10 g/L, yeast extract 5 g/L, K_2_HPO_4_ 2 g/L, anhydrous sodium acetate 5 g/L, ammonium citrate 2 g/L, MgSO_4_·7H_2_O 0.58 g/L, MnSO_4_·H_2_O 0.25 g/L, Tween-80 1 mL/L) broth or agar medium at 30 °C. *Escherichia coli* DH5α was used as an intermediate cloning host and was grown at 37 °C with agitation in Luria Broth (LB) broth. Antibiotics were used at the following concentrations for *E. coli*: erythromycin (Em) 150 μg/mL; chloramphenicol (Cm) 15 μg/mL. For *Leu. mesenteroides* DRP105, the concentrations were as follows: Em, 5 μg/mL; Cm, 5 μg/mL. The plasmid pNZ5319 was used to construct knockout vectors. The *cre* gene expression plasmid pNZTS-Cre was constructed according to the method of Zhu et al. [20].

### 3.2. Phylogenetic Analysis

*Leu. mesenteroides* DRP105 was cultured in MRS medium at 30 °C, and the genomic DNA was extracted using a PrimeScript™ RT reagent (Takara Bio, Beijing, China), following the standard protocol recommended by the manufacturer. The 16S ribosomal DNA (rDNA) gene of DRP105 was amplified using the universal primers 27F and 1492R [23]. On the basis of the results of homologous gene analysis, the sequences of the reference strains used for phylogenetic analysis were obtained from the Multiple Alignment with Fast Fourier Transform (MAFFT) (http://mafft.cbrc.jp/alignment/software/) databases. The comparison quality control was conducted using Gblocks software (http://molevol.cmima.csic.es/castresana/Gblocks.html). A phylogenetic tree was generated using the maximum-likelihood method with RAxML software (https://github.com/stamatak/standard-RAxML) [24].

### 3.3. Genome Sequencing and Assembly

The genomic DNA of *Leu. mesenteroides* DRP105 was sequenced using the PacBio RS II platform with a 300-bp paired-end library by Majorbio Bio-pharm Technology Co., Ltd. (Shanghai, China). The reads were assembled using CLC Genomics Workbench 6.0 (CLCbio). The initial assembly was converted for the CLC Genomics Workbench by constructing artificial reads from the consensus to collect the read pairs in the PacBio paired-end library. CodonCode Aligner 3.7.1 (CodonCode Corp.) was used for the sequence assembly and quality assessment in the subsequent finishing process [25].

### 3.4. Genome Annotation

Using the Rapid Annotations Subsystems Technology (RAST) online server (http://www.rast.nmpdr.org/) to predict protein coding sequences (CDS), we completed the functional annotation of the predicted genes and identification of tRNA and rRNA [26]. The Gene Ontology (GO) [27], KEGG [28], COG [29], Nonredundant Protein Database (NR), Swiss-Prot, and carbohydrate-active enzymes [30] databases were used to predict gene functions. A genome-wide BLAST search was conducted against the above seven databases (with the E-value less than 1 × 10^−5^ and the minimum alignment length percentage greater than 40%). The genome sequence of this strain was obtained from the EzGenome database (http://ezgenome.ezbiocloud.net) and used to calculate the ANI value [15].

### 3.5. Dextransucrase Gene Analysis

The translated amino-acid sequence of the *dsr* gene was obtained using DNAMAN software. The secondary structure and three-dimensional (3D) model of Dsr were produced using PSI-PRED (https://npsa-prabi.ibcp.fr/cgi-bin/npsa_automat.pl?page=npsa_sopma.html) and SWISS-MODEL implementation (http://swissmodel.expasy.org/), which uses the MODELLER program [31].

### 3.6. Construction of the dsr Gene Knockout Vector

To construct the *dsr* gene knockout vector, standard cloning procedures were used. Typically, about 1.0-kb fragments of 5′ (primers *dsr*-up-F CGCCTCGAGGGCTTAGATAATCAAGATG, *dsr*-down-F CGCCCCGGGGCTTTCTGTGCATATAGTG were used) and 3′ (primers *dsr*-up-R CGCGTTTAAACAAATTTTCTCCTCATAATATTC, *dsr*-down-R CGCGAGCTCGTTAATTTGTGCCATACCAT were used) flanking regions of *dsr* gene were amplified with the *Leu. mesenteroides* DRP105 chromosome as a template for PCR using a proof-reading polymerase and ligated into the *Xho*I-*Pme*I and *Sma*I–*Sac*I restriction sites of pNZ5319, respectively. They were then transformed into the *E. coli* DH5α strain. By following the above strategy, the pNZ5319 *dsr* KO vector (pNZ5319−Δ*dsr*) was constructed.

### 3.7. Mutant Construction

We used the following strategies for constructing *dsr*-deficient strains and the subsequent removal of the cm cassette by Cre-loxP: the pNZ5319−Δ*dsr* vector was transformed into *Leu. mesenteroides* DRP105 competent cells with electroporation. Then, 50 mL of competent cells and 2 μL (80 ng) of pNZ5319−Δ*dsr* plasmids were mixed and transferred into an ice-cooled electroporation cuvette (0.1 cm electrode gap). After electroporation, the suspension was immediately mixed with MRS medium and incubated at 30 °C for 4 h, and then the cells were cultured on MRS plates containing Cm. After 36 h, Cm^r^ transformants were selected and plated again to check for an Em^s^ phenotype [20]. In order to identify the single and double crossover recombinants, the transformants were used as templates for the amplification with the primers Cm-F ATGAACTTTAATAAAATTGATTT, Cm-R TTATAAAAGCCAGTCATTAGG, Ery-F AAAAATAGACAATACTTGCTCATAA, Ery-R ATTTAAAAGAAACCGATACCG, *dsr*-1-F CCAATTGACTGTTTTTACAATG, *dsr*-1-R GATAAATACTATAATCAAGT, and 4-F, 4-R. The PCR products were analyzed on 1% agarose gels.

### 3.8. Cre-Mediated Mutant Locus Resolution

The clean knockout genotype of strains was made by electroporating the pNZTS-Cre plasmid into *Leu. mesenteroides* DRP105−*Δdsr* competent cells. Then, we picked Emr colonies and inoculated them in MRS containing Em at 30 °C overnight and then inoculated 1% in 5 mL of MRS containing Em 10 times before finally inoculating at 30 °C with Cm and Cm-Em on an MRS plate. After that, Cms-Emr transformants were selected and incubated at 42 °C for 2 h, then moved to 30 °C for 24 h, and then 50 μL of a 10^−5^ dilution was spread on MRS plates to cultivate without antibiotic medium at 30 °C [32]. One hundred colonies were then transferred to MRS plates containing Em with toothpicks and incubated at 30 °C. *Leu. mesenteroides* DRP105−*Δdsr* was detected by PCR using TGGTATCCAATTTACTGACCG and CATTGCCCCTGTTTCACTAT primers. Then, the *dsr*-null strain was selected and stored as a glycerol stock solution.

### 3.9. Assay of Growth Profiles

The *Leu. mesenteroides* DRP105 and *Leu. mesenteroides* DRP105−*Δdsr* were inoculated into MRS-S medium (sucrose 20 g/L, tryptone 10 g/L, beef extract 10 g/L, yeast extract 5 g/L, K_2_HPO_4_ 2 g/L, anhydrous sodium acetate 5 g/L, ammonium citrate 2 g/L, MgSO_4_·7H_2_O 0.58 g/L, MnSO_4_·H_2_O 0.25 g/L, and Tween-80 1 mL/L) and cultured with shaking at 30 °C. Every 6 h, 1 mL of fermentation broth was 10-fold diluted. We centrifuged 1 mL of the diluted solution at 11,000× *g* for 10 min. The supernatant was filtered through a 0.45-μm filter membrane. The dextran content was determined using the phenol sulfuric acid method [33]. The sucrose, glucose, and fructose contents were determined using ion chromatography [34]. The citric acid, pyruvic acid, succinic acid, formic acid, lactic acid, malic acid, acetic acid, and α-ketoglutaric acid contents in the fermentation broth were determined using high-performance liquid chromatography (HPLC) (Alliance2695, Water, Milford Massachusetts, USA) [35]. The content of free amino acids at 24 h was determined using an automatic amino-acid analyzer (S-433D, Sykam, Munich, Germany) [36].

### 3.10. Nucleotide Sequence Accession Numbers

The complete genome of *Leu. mesenteroides* DRP105 was deposited in NCBI under the accession number SRR12201911.

### 3.11. Statistical Analysis

The data are presented as the mean ± standard errors of three independent experiments. The statistical data were analyzed using JMP software (SAS Institute Inc., Version 9.0.2). The differences between the sets were compared using ANOVA software. The differences were defined as significant when the *p*-value was <0.05.

## 4. Conclusions

In this study, we determined the complete genome sequence of *Leu. mesenteroides* DRP105 with the PacBio RS II MiSeq platform, and a *dsr* gene was selected. The Dsr coded by the *dsr* gene has the highest similarity to the *Leu. mesenteroides* Dsr (ABC75033.1). It belongs to the GH70 family and contains eight conserved regions. The structure of the enzyme protein translated from the *dsr* gene was consistent with that of Dsr. A *dsr* gene-deficient strain derived from *Leu. mesenteroides* DRP105 was first produced using the Cre-loxP recombination system. The result showed that the *dsr* gene plays a key role in the sucrose metabolism. This changed the sucrose metabolic pathway of *Leu. mesenteroides* DRP105 and affected the production of dextran. These results provide valuable information for strain engineering based on genetic information and provide a theoretical basis for further revealing the biosynthesis mechanism of LAB dextran.

## Figures and Tables

**Table 1:** Statistics table of PacBio raw data.

Statistics of PacBio Raw Data	*Leu. mesenteroides* DRP105
Total read number	297,458
Total bases (bp)	2,715,405,332
Largest (bp)	49,431
Average length (bp)	9218

**Table 2:** Statistics table of the gene prediction information.

Sample	Chromosome	Plasmid-DRP01	Plasmid-DRP02	Plasmid-DRP03	Plasmid-DRP04	Plasmid-DRP05
No. of all scaffolds	1	1	1	1	1	1
Bases in all scaffolds (bp)	1,815,345	37,314	13,823	12,703	12,404	12,703
Scaffolds N50 (bp)	1,815,345	37,314	13,823	12,703	12,404	12,703
G + C content (%)	37.267	35.388	36.743	35.558	35.415	37.967
N rate (%)	0	0	0	0	0	0
Gene number	1804	49	20	17	11	12
Gene total length (bp)	1,597,347	24,591	10,311	9690	10,167	4989
Gene average length (bp)	885	520	515	570	924	415
Gene density (/kb)	0.993	1.313	1.446	1.338	0.886	0.944
GC content in gene region (%)	37.8	36.5	38.2	35.9	35.6	33.2
Gene/Genome (%)	88.0	68.3	74.6	76.3	82.0	39.3
Intergenetic region length (bp)	217,998	11,823	3521	3013	2337	7714
GC content in intergenetic region (%)	32.8	32.8	32.2	34.2	34.1	41.0
Intergenetic length/genome (%)	12.0	31.7	25.4	23.7	18.0	60.7

**Table 3:** The free amino-acid content in *Leu. mesenteroides* DRP105 and *Leu. mesenteroides* DRP105−*Δdsr* at 24 h.

Amino Acids	Content (mg/kg)		*p*-Value
	*Leu. mesenteroides* DRP105	*Leu. mesenteroides* DRP105−*Δdsr*	
Tau	0 ± 0	0±0	1
Asp	56.0 ± 1.1	56.2 ±1.4	0.1056
Thr	82.9 ± 1.4	82.4 ± 0.6	0.0194
Ser	68.0 ± 2.3	64.5 ± 1.9	0.0004
Glu	42.5 ± 1.0	32.4 ± 0.4	<0.0001
The	0 ± 0	0 ± 0	1
Gly	200 ± 4.8	202 ± 3.5	0.0012
Ala	231 ± 3.1	238 ± 1.8	0.0001
Cys	24.7 ± 0.3	26.2 ± 1.1	0.0022
Val	159 ± 1.6	160 ± 2.1	0.0050
Met	89 ± 0.7	87 ± 1.5	0.0012
Ile	120 ± 2.3	115 ± 3.4	0.0002
Leu	515 ± 1.5	506 ± 1.3	<0.0001
Tyr	296 ± 2.3	287 ± 2.5	<0.0001
Phe	341 ± 4.1	338 ± 3.7	0.0006
g-ABA	15.9 ± 0.2	15.7 ± 1.1	0.1056
Lys	283 ± 2.7	282 ± 3.8	0.0050
His	70.6 ± 2.1	66.1 ± 1.2	0.0002
Arg	687 ± 4.5	681 ± 3.6	0.0001
Hypro	0 ± 0	0 ± 0	1
Pro	48.1 ± 1.7	44.2 ± 2.1	0.0003
Total	3329.7 ± 5.3	3283.7 ± 3.8	<0.0001
